# Application of an Electronic Nose for Early Detection of Tephritidae Infestation in Fruits

**DOI:** 10.3390/insects17040429

**Published:** 2026-04-16

**Authors:** Eirini Anastasaki, Aikaterini Psoma, Mattia Crivelli, Savina Toufexi, Maria-Vassiliki Giakoumaki, Panagiotis Milonas

**Affiliations:** 1 Scientific Directorate of Entomology and Agricultural Zoology, Benaki Phytopathological Institute, 14561 Kifissia, Greece; e.anastasaki@bpi.gr (E.A.); a.psoma@bpi.gr (A.P.); s.toufexi@bpi.gr (S.T.); m.giakoumaki@bpi.gr (M.-V.G.); 2 PCA Technologies S.r.l., 20005 Pogliano Milanese, Milan, Italy; m.crivelli@pcatechnologies.com

**Keywords:** e-nose, fruit flies, *Ceratitis capitata*, *Bactrocera dorsalis*, *Bactrocera zonata*, chemometric, KNN

## Abstract

Identifying pest infestations in fresh fruits is a crucial aspect of international trade. Currently, inspections rely on visual observations and destructive sampling, which are, in most cases, quite demanding. The detection of oviposition signs or early larval development is largely not feasible. Therefore, new methods that are sensitive and non-destructive are urgently needed to detect fruit fly infestation during inspections of fresh produce before their introduction and spread into pest-free areas. Portable electronic olfactory systems, or electronic noses (e-noses), are used in various scientific fields and industries. In this study, we evaluated the potential of a portable PEN3 electronic nose to discriminate between non-infested and infested fruits for three fruit fly species: *Ceratitis capitata* (Wiedemann), *Bactrocera dorsalis* (Hendel), and *Bactrocera zonata* (Saunders) (Diptera: Tephritidae). E-nose datasets were generated from samples of each combination of fruit, fruit fly species, infestation status, and storage condition. These datasets were used to develop classification models. The classification accuracy of the models ranged from 50 to 99% during calibration and cross-validation conditions. However, their performance decreased substantially when applied to independent datasets, highlighting limitations in robustness. These findings indicate that although the PEN3 system shows promise as a non-destructive detection tool, its performance is strongly influenced by seasonal and experimental variability. Further work is needed to incorporate multi-season and multi-variety datasets, improve calibration, and robust validation before practical implementation in field inspection systems.

## Simple Summary

Detecting pest infestations in fresh fruits is vital for preventing the spread of invasive species through international trade. Current methods rely on visual inspection and destructive sampling, which are often ineffective for detecting early-stage infestations. In this study, we evaluated the use of the portable PEN3 electronic nose (e-nose) as a potential rapid and non-destructive approach to distinguish between non-infested and infested fruits for three fruit fly species: *Ceratitis capitata*, *Bactrocera dorsalis*, and *Bactrocera zonata*. The e-nose analyzed headspace volatiles emitted from various fruit samples under different storage conditions to build a comprehensive volatile fingerprint database. Results demonstrated that the PEN3 e-nose accurately distinguished between infested and non-infested fruits under controlled conditions. However, performance decreased substantially when models were applied to independent datasets, indicating limitations in robustness. These findings suggest that the method has potential as a sensitive, rapid, and non-destructive tool. However, further validation across multiple seasons and conditions is required before it can be reliably used in quarantine inspections and pest management.

## 1. Introduction

Tephritid fruit flies are among the most devastating insect pests, causing significant yield losses for the fresh fruit production industry worldwide [1]. Within Europe, horticultural systems are increasingly threatened by several established species, such as *Ceratitis capitata* (Mediterranean fruit fly), and regulated quarantine species, such as *Bactrocera dorsalis* (oriental fruit fly) and *Bactrocera zonata* (peach fruit fly) [2,3]. While *C. capitata* is already established in warmer Mediterranean regions and is spreading northward [4], *B. dorsalis* and *B. zonata*, both of Asian origin, are not yet established in mainland Europe. However, incursions have been observed, and these species may rapidly spread into Europe and become established, favored by climate change. Recent incursions in Europe [5] emphasize the urgent need for rapid, sensitive, and non-destructive detection technologies during border inspections. Strict quarantine regulations are in place to prohibit the introduction of invasive fruit flies in new areas. Currently, border inspections for infestations rely on visual observations and destructive sampling. For example, for citrus inspection, the EPPO Standard PM3/90(1) [6] recommends that a minimum of 300 fruits be inspected with destructive sampling to obtain an acceptable level of pest-free status in the consignment. This is obviously time-consuming and labor-intensive. Moreover, the effectiveness of visual observations depends considerably on the development of pest-specific symptoms. However, this is not always the case with fruit flies, where symptoms appear in the late phase of infestation. The detection of oviposition signs or early larval development is practically not feasible [7,8].

Early detection of fruit fly infestations is critical for ensuring the accuracy and efficiency of pest detection, particularly within import control procedures. Non-destructive methods have gained significant attention for fruit fly infestation. Among these, near-infrared (NIR) spectroscopy has emerged as a promising technique for detecting insect infestation in fruits [9]. It has been used for the early detection of *Bactrocera oleae* infestation in olive fruits [10] and *Ceratitis capitata* infestation in orange fruits [11], as specific spectral bands contain information on various molecular vibrations and functional groups. In addition, technologies such as thermal or hyperspectral imaging have demonstrated considerable potential for identifying infestations [11]. However, these technologies are still in an experimental status as several barriers hamper the implementation in the field [12].

Another approach for non-destructive inspection of commodities for pest detection is the use of volatile organic compounds (VOCs) emitted by plants. VOCs emitted by infested fruits have previously been investigated as indicators of fruit fly activity. The VOC emission profile of host fruits is modified as a response to herbivory [7,13,14,15,16,17], and the new profile may reflect oviposition and larval development stages [13]. Headspace techniques, either static, like solid-phase microextraction (SPME) [13,18,19] and/or dynamic [7,15,16,17,20] in combination with gas chromatography–mass spectrometry (GC-MS), have been widely used for the collection, detection, and identification of fruit VOCs. Although these techniques are non-destructive, very sensitive, and accurate, they are expensive and time-consuming, and they require specialized staff and equipment.

An alternative approach is the use of an electronic nose (e-nose) for VOC-based detection. An electronic nose employs an array of chemical sensors with broad selectivity, which respond to diverse VOCs and, through recognition algorithms, can discriminate among complex odors [21,22]. Once trained with representative datasets, an e-nose can perform rapid classification tasks in real time. Among commercially available systems, the PEN3 e-nose (Airsense Analytics) uses metal oxide semiconductor (MOS) sensors and has been successfully applied in areas such as food quality [23,24], environmental monitoring, water quality assessment [25], and medical diagnostics [26,27]. Applications of e-noses in plant sciences have mainly focused on detecting bacterial, fungal, and viral infections [27,28,29,30,31,32], with only limited work on insect pest detection [33,34,35,36,37,38,39,40]. Previous studies have used e-nose technology to detect insect infestation, including stink bugs in cotton [35], the rice stem borer *Chilo suppressalis* (Walker) and the brown planthopper *Nilaparvata lugens* (Stål) in rice plants [36], the rice weevil *Sitophilus oryzae*, red flour beetle *Tribolium castaneum* and lesser grain borer *Rhyzopertha dominica* in stored rice [37], the lesser grain borers *Rhyzopertha dominica* (F.) in wheat [38], *Ephestia kuehniella* in white flour [39], *Myzus persicae* in tomato plants [40], and *B. dorsalis* infestation in citrus [33]. However, many of these studies often used custom-built sensor arrays [33,35,39,40] or other e-nose systems [37,38], lacking external validation [35,38,39], or were conducted under tightly controlled laboratory conditions [37,39], without addressing cross-seasonal variability. All the above do not target specific fruit and fruit fly species combinations and lack validation with independent datasets.

The present study is the first to apply a commercially available metal oxide semiconductor (MOS) gas sensor type electronic nose device (the PEN3 e-nose) to detect infestation of three fruit fly species, *Ceratitis capitata* (Wiedemann), *Bactrocera dorsalis* (Hendel) and *Bactrocera zonata* (Saunders) (Diptera: Tephritidae), across multiple fruit hosts (peach, apple, orange, and mandarin), storage conditions, and season variability. The developed models were also validated on independent datasets to obtain robust validation.

## 2. Materials and Methods

### 2.1. Insect Rearing

#### 2.1.1. *Ceratitis capitata* (Mediterranean Fruit Fly or Medfly)

A laboratory colony of *C. capitata* was established in 2013 at the premises of the Benaki Phytopathological Institute (BPI) (Kifissia, Greece) and has been renewed each year using wild individuals collected from bitter oranges in the area of Attica, Greece. The colonies of flies were kept under laboratory conditions at 25 ± 1 °C, 55–65% relative humidity, and a 14 h light–10 h dark photoperiod.

#### 2.1.2. *Bactrocera dorsalis* and *Bactrocera zonata*

Laboratory rearings of *Bactrocera dorsalis* and *Bactrocera zonata* were established in a quarantine greenhouse biosecurity facility in December 2019, with pupae received by Dr Hélène Delatte (CIRAD), UMR “Peuplements Végétaux et Bio-agresseurs en Milieu Tropical”, La Réunion, France. The colonies of flies were kept under laboratory conditions in the quarantine greenhouse biosecurity at 25 ± 1 °C, 55–65% relative humidity, and a 14 h light–10 h dark photoperiod.

### 2.2. Fruit Infestation

Four different fruits were tested, namely, peaches (*Prunus persica* var. *nucipersica*), apples (*Malus domestica*), variety Starking delicious, mandarins (*Citrus reticula*), variety clementine, and two varieties of oranges (*Citrus sinsensis*): Washington navel and Valencia. The following procedure was followed to obtain fruits with oviposition stings by each fruit fly species. In a cage (60 × 60 × 60 cm) (Bugdorm, Taichung, Taiwan) covered by organdy gauze, 100 mated adult flies (16 to 20 days old) [41] were placed at a sex ratio of 1:1. All three fruit fly species were maintained with ad libitum access to sugar, protein, and water. Depending on fruit texture and fruit fly developmental time and survival under the different storage conditions used, we established a specific protocol for oviposition and data acquisition for each fruit.

Peaches were obtained from a local food market in summer 2021, infested by *B. zonata* and *C. capitata*, and tested under two different storage conditions. Ten fruits were placed in the cage and removed after 2 h each time. Infested fruits and controls (fruit never exposed to fruit flies) were kept under laboratory conditions at 25 ± 1 °C and 20 ± 1 °C. Data acquisition was performed immediately (d0-eggs), 24 h (d1-eggs), 4 days (d4-L1 to L2) and 5 days (d5-L1 to L2) after oviposition for fruit storage at 25 °C and immediately (d0-eggs), 24 h (d1-eggs) and 7 days (d7-L1) after oviposition at 20 °C. Control non-infested fruits were also sampled at the same time intervals after oviposition. It was estimated that 500–700 g of peaches was used in each sample (3 peaches per sample).

For the infestation of apples by *C. capitata* and the two *Bactocera* species, ten fruits were placed in the cage and removed after 24 h each time. Fruits were supplied by the Agricultural Cooperative of Zagora Zagorin (Volos, Greece). Fruits (infested and control) were stored at 6 ± 1 °C. Data acquisition was performed 1 (d0-eggs) and 15 (d15-eggs) days after oviposition. Control non-infested fruits were also sampled at the same time intervals after the oviposition. In the case of apples, one apple per sample was used.

Oranges and mandarin fruits were supplied by G.N. Fragistas S.A. (Argos, Argolida, Greece). Seven orange fruits and twelve mandarin fruits were placed in the cage and removed after 2 h each time. Infestation by all fruit fly species was performed for orange fruits, while for mandarin fruits, infestation by *C. capitata* and *B. zonata* was performed. Fruits (infested and control non-infested) were stored at 25 ± 1 °C. Data acquisition was performed immediately (d0, eggs), 24 h (d1, eggs), and 8 days (d8, L2) after oviposition. It was estimated that 300–400 g of mandarins was used (3 fruits per sample). One orange per sample was used.

Due to time and space limitations, fruit infestation by fruit flies was carried out in several batches of fruits to obtain a complete dataset. After oviposition, all fruits were inspected under a stereoscope for oviposition stings. Following data acquisition at the final treatment, the fruits were inspected again under a stereoscope to confirm the presence of larvae. In all cases, at least 45–60 replicates per treatment were performed.

### 2.3. Sample Preparation and E-Nose Data Acquisition

Analyses were performed using the PEN3 electronic nose (Airsense Analytics GmbH, Schwerin, Germany). PEN3 consists of a gas sampling unit, a detector unit with a sensor array, and pattern recognition software (Win Muster v.1.6.2.23). The sensor array comprises 10 different metal oxide gas sensors (MOSs). The sensor characteristics are shown in Table S1. Before the experiment, the sensors were preheated to achieve stable conditions. MOSs are sensitive to a broad range of volatile chemical compounds due to different operating temperatures within the range 150–500 °C. The reaction mechanism is based on the exchange of oxygen between volatile molecules and the metal layer, resulting in a change in electrical resistance that is related to the nature of the adsorbed compound. The change in electrical conductivity is detectable by electrodes attached to each sensor. The pattern recognition software gives a graphic display of relative sensor values.

Based on preliminary trials performed for the optimization of e-nose data acquisition, the operating conditions were adjusted as follows: pre-sampling time 10 s, measurement time 80 s, flush time 100 s, zero-point count auto 10 s, chamber flow 400 mL/min, and injection flow 400 mL/min. Each fruit sample was enclosed in polyethylene terephthalate (PET) oven-roasting bags with a thickness of 12 μm (SANITAS, Sarantis Group, Athens, Greece) and maintained at a room temperature of 25 ± 1 °C for 15 min to allow the accumulation of VOCs emitted from the fruits. The headspace volatiles were then pumped into the sensor chamber at a constant flow rate of 400 mL/min, resulting in a total detection time of 200 s per sampling cycle. Before each measurement, a cleaning procedure for the sensor array was performed for 100 s, and the baseline was adjusted after a zero-point count for 10 s. Following a 10 s pre-sampling time, the probe was inserted into the oven bag, and measurements were recorded for 80 s.

### 2.4. Data Analysis

The sensor-array response outputs for the e-nose dataset were used for analysis, focusing on the stable signal range between 60 and 80 s. Raw signals acquired from the PEN3 electronic nose were subjected to a structured preprocessing workflow prior to statistical analysis. Baseline correction was applied using the initial phase of each measurement cycle (zero-point acquisition) to reduce background signal variability. Subsequently, feature extraction was performed by selecting the stabilized response of each MOS at the end of the measurement phase, which represents the odor fingerprint of each sample. For each repetition, the dataset was defined using the sensor values at 80 s, corresponding to the plateau of each sensor. The resulting dataset was then normalized using auto-scaling, ensuring comparability among sensors with different sensitivities and dynamic ranges. Signal stability was evaluated within each measurement session to assess potential drift effects. Due to the relatively short acquisition time and controlled experimental conditions, no significant drift requiring correction was observed. Outlier detection was carried out using a combined approach. First, sensor response curves were visually inspected to identify anomalous or unstable measurements. Second, multivariate statistical analysis was performed using principal component analysis, and samples falling outside the confidence limits were considered potential outliers. Such samples were excluded from further analysis only when the anomalous behavior could be attributed to instrumental instability or sampling irregularities.

**Figure 1:**
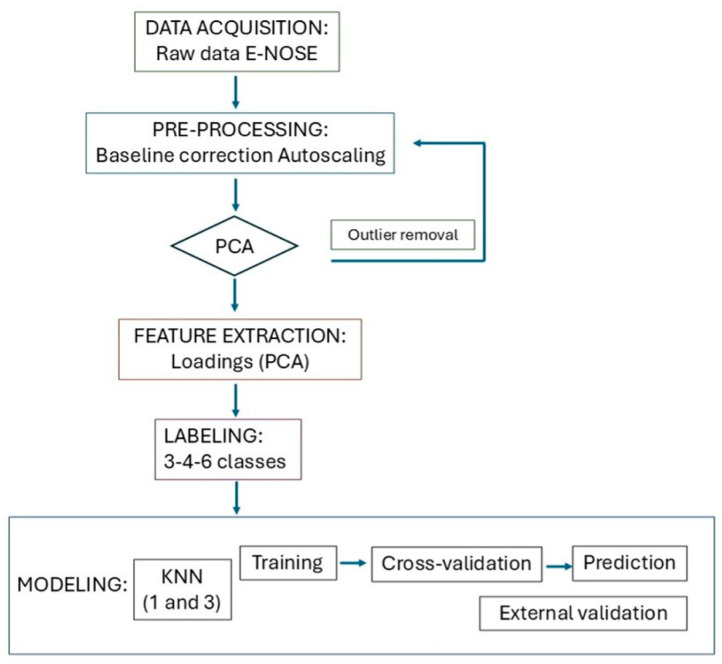
Schematic representation of the methodology.

After the outlier removal procedure, if needed, the data were auto-scaled again to overcome scale differences related to different days of analysis and sensor response ranges. PCA was performed on the cleaned dataset. Classification models were developed using the K-Nearest Neighbor (KNN) algorithm, evaluating different values of k and group classes (Table 1). The reliability of each classification strategy (model) was tested by performing calibration and cross-validation chemometric analyses on the training dataset. Then, to test the real capability of the models to predict new samples, each dataset was split into two subsets: 70% of the data were used for calibration and internal validation (cross-validation), whereas the other 30% were removed and used in a second step to check the capability of the model when predicting samples that were not used in the calibration phase. After model construction, external validation was performed for the evaluation of the models. All preprocessing and statistical analyses were performed using MATLAB (MATLAB R2024b version, PLS toolbox 9.5), ensuring consistency and reproducibility of the data processing workflow. An overall schematic representation of the methodology is illustrated in Figure 1. The “R language” was also used to illustrate the PCA figures and loading plots.

## 3. Results

### 3.1. Peaches

**Figure 2:**
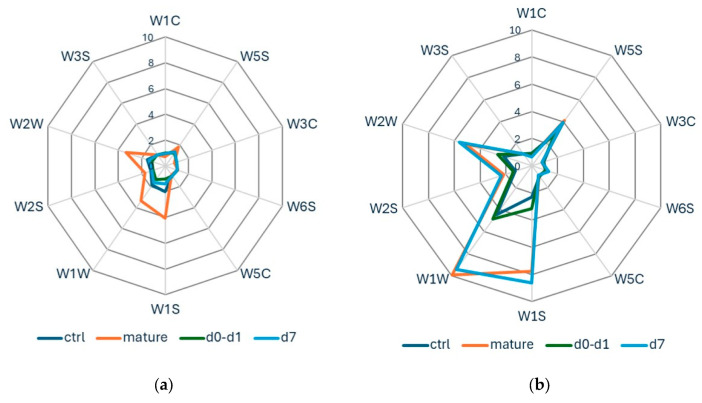
Radar plots of e-nose sensor response values for (**a**) peaches after infestation by *B. zonata*, (**b**) peaches after infestation by *C. capitata*, stored at 20 °C, and their corresponding control samples.

All ten sensors of the PEN3 portable E-nose exhibited varying degrees of response (Figure 2). The W1W and W1S sensors had a high average response, followed by W2W and W5S. W1C, W3C, and W5C had average responses below 1.

**Figure 3:**
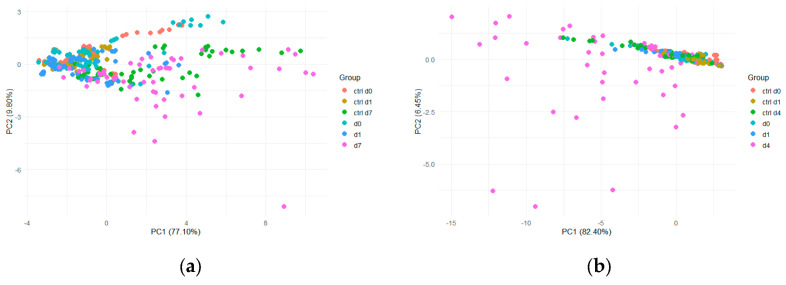
PCA score plots using the first two PCs (principal components) for all peach samples analyzed and divided into six classes for: (**a**) E-nose data acquired after infestation by *C. capitata* and stored at 20 °C; (**b**) E-nose data acquired after infestation by *B. zonata* and stored at 25 °C.

PCA was initially performed on all samples to evaluate possible differentiation according to the infestation level and the maturity of the samples (Figure 3). PCA did not allow for a clear separation between sampling time and non-infested or infested samples. However, a higher dispersion of infested samples was observed after 4 days (d4, yellow dots) compared to the other group of samples, especially for *B. zonata* stored at 20 °C (Figure 3b).

In Table 2, the percentage of correct classification rates for calibration (cal), cross-validation (CV), and prediction for all peach models for *B. zonata* and *C. capitata* at two storage temperatures are shown.

*B. zonata*

The initial dataset, generated after infestation and storage at 25 °C, consisted of 335 samples distributed among six classes (Table 1). After outlier removal, 279 samples remained and were used for model development, which were further divided into the training and prediction sets. When classified into six classes, the overall classification capability was 57% for K distances 1 and 3 (Table 2). When the number of classes was reduced to four, a noticeable improvement in classification performance was observed. The overall accuracy increased to 59% for k = 3 and 65% for k = 1 (Table 2), indicating that class consolidation enhanced model robustness.

For the dataset at 20 °C, the initial dataset, generated after infestation and storage at 25 °C, consisted of 257 samples (Table 1). After outlier removal, 181 samples remained, which were further divided into the training and prediction sets. The model achieved an overall classification of 67% when using six classes for both K distances 1 and 3. When the number of classes was reduced to four, model performance improved, giving an overall correct classification rate of >75% (Table 2). Evaluation of the prediction set demonstrated that the model with K = 1 can be considered applicable. Especially in the K = 1 model, the overall rate reached 85%.

*C. capitata*

For the *C. capitata* dataset stored at 25 °C, the initial dataset, consisting of 311 samples (Table 1), was reduced to 226 samples after outlier removal. The overall classification rates on the prediction set for six classes were slightly above 60% for K = 3 and 71% for K = 1. As observed in the case of the *B. zonata* dataset, reducing the number of classes to four improved model performance. The overall classification capability on the prediction set was 69% for K distance 3 and 75% for K = 1.

At 20 °C, the initial dataset consisted of 350 samples (Table 1). Following outlier removal, the dataset was reduced to 291 samples, which were divided into the training and the prediction set. The overall classification capability for the six-class models was 71% for both K values examined (K = 1 and K = 3). When the models were developed using four classes, the performance improved, and the overall classification reached 80% for K distance 3 and 85% for K = 1. The latter model seems to be robust enough to work properly in prediction.

External validation

The models with KNN = 1 at 20 °C seemed to be robust enough to be further used for an external validation set, using samples from different varieties or cultivation years. The validation procedure was performed using the models developed with the dataset from 2021; an independent new dataset was collected in summer 2022, for each of the following conditions: control d0, control d1, control d7, d0, d1, and d7. However, comparing the sensor signals obtained from this dataset with those from the calibration–cross-validation phase, a substantial gap between signals was observed, making it impossible to complete the validation procedure.

In summer 2023, a new dataset of measurements was acquired, aiming to validate the predictive models previously developed for peaches infested by *C. capitata* and *B. zonata* and stored at 20 °C, since the feedback from the initial validation of the model was not satisfactory. Applying the new dataset to the model built during the summer of 2021, the overall classification rate of *B. zonata* was 49%. The model could predict satisfactorily (95%) only for infested samples collected immediately after oviposition (d0) and one day after oviposition (d1). For the three other classes, correct classification was decreased to 42% for infested peach fruits collected 7 days after oviposition and 17% for the control samples. For *C. capitata* infestation, the overall classification was 43%, and the model correctly predicted 87% of the infested samples collected immediately after oviposition (d0) and 1 day after oviposition (d1). Accuracy for later stages and control samples was considerably lower (Supplementary Materials). However, the majority of false predictions were for false positives.

### 3.2. Apples

**Figure 4:**
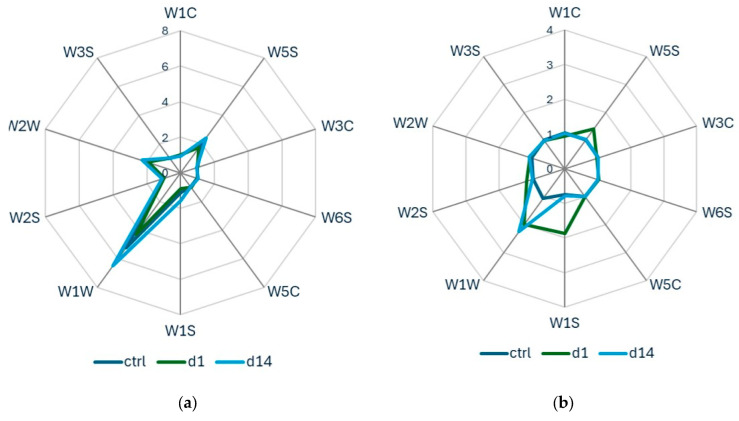
Radar plots of e-nose sensor response values for apples after infestation by *B. dorsalis* and their corresponding control samples (**a**) in 2022 and (**b**) in 2023.

**Figure 5:**
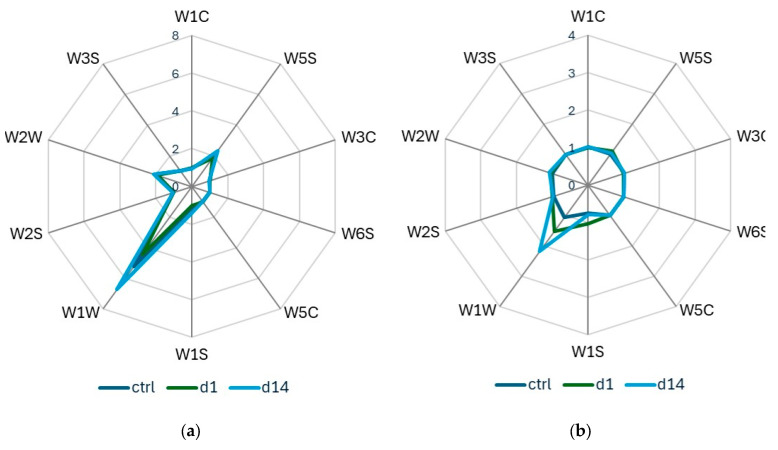
Radar plots of e-nose sensor response values for apples after infestation by *C. capitata* and their corresponding control samples (**a**) in 2022 and (**b**) in 2023.

**Figure 6:**
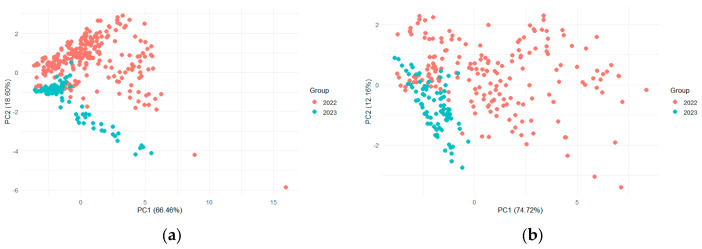
PCA score plots using the first two PCs (principal components) for all apple samples classified according to the year acquired for: (**a**) E-nose data after infestation by *B. dorsalis* and (**b**) E-nose data after infestation by *C. capitata*.

For apple fruits, samples were stored at 6 °C, and data were collected during two consecutive years (2022 and 2023) for *B. dorsalis* and *C. capitata*. For *B. zonata*, e-nose data acquisition was performed only for the year 2022. Analysis of the radar plots of the E-nose responses revealed that all apples exhibited high signal intensities on sensor W1W when infested by *B. dorsalis* (Figure 4). However, signal intensities showed considerable variation between the two sampling years. In the first year, 2022, both the control and infested apple samples exhibited a common sensor pattern (Figure 4a). Yet, in the second year, the sensor responses varied across the control and infested samples (Figure 4b). A similar tendency was observed for *Ceratitis capitata* (Figure 5), with the ten sensors showing varied responses over the two-year period. This is also shown in Figure 6, where the PCA score plots for all samples infested by *B. dorsalis* and *C. capitata* over the two years indicate a large variation in sensor intensities.

During 2022, models were constructed without applying outlier removal. Models with KNN 1 and 3 were developed by classifying the samples into either four or three classes. Table 3 shows the percentage of correct classification rates for calibration (cal), cross-validation (CV), and prediction for all models constructed for *B. dorsalis*, *B. zonata*, and *C. capitata.*

*B. dorsalis*

The dataset used for *B. dorsalis* models consisted of 254 samples and demonstrated high predictive performance, with classification accuracy exceeding 80%. Specifically, for the four-class model with a K-nearest neighbor (KNN) value of 3, the overall classification rates for calibration, cross-validation, and prediction were all above 80%. When employing a model with KNN = 1, which corresponds to the shortest comparative distance, the classification rates were improved. The overall prediction accuracy reached 88%, with individual class accuracy ranging between 86% and 90%. Further improvement was observed using a three-class model. In the case of KNN = 3, the overall prediction accuracy of KNN = 1 was applied; the prediction accuracy improved further to 91%, with individual class accuracy between 86% and 89%.

*B. zonata*

For *B. zonata* models, a total of 201 samples was used. The four-class model achieved an overall prediction accuracy of 69%. A three-class model provided a marginal improvement over the four-class model.

*C. capitata*

The dataset for *C. capitata* consisted of 237 samples. Models using KNN = 1 yielded predictive performance values of 85 and 86% for the four- and three-class models, respectively. Models with KNN = 3 provided up to 80% prediction accuracy.

Given that the above models were consistent and had sufficient prediction capacity, a validation procedure was planned to be carried out for the models developed for *B. dorsalis* and *C. capitata*. New datasets were acquired for both aforementioned species for the year 2023. The outlier removal approach was used, and the overall accuracies of the models that were constructed are shown in Table 4.

*B. zonata*

The initial dataset consisted of 120 samples (Table 1). After outlier removal, 78 samples were used for model calibration and prediction. Notably, when the three-model class approach was used, the model had high classification rates, and the prediction reached an accuracy of 92% for both KNN values tested.

*C. capitata*

The dataset for *C. capitata* consisted of 108 samples. Following outlier removal, the dataset consisted of 85 samples. Three-class models yielded a predictive performance of 97% for both KNN values.

Prior to external validation, after comparing the sensor signals from the data used for model construction and prediction for the year 2022, the gap between signals was so wide that it was not possible to complete the validation procedure. Therefore, new models were developed by combining data from both years to broaden applicability across sampling years and overcome this constraint. The initial dataset for *B. dorsalis* consisted of 374 samples, which were reduced to 258 after outlier removal. The models achieved calibration and cross-validation accuracy rates fluctuating between 91 and 95% across all combinations of classes and KNN values (Table 5). However, the prediction capability of the model was very low, yielding success rates of 47% for the three-class models and only 21% for the four-class models (Table 5).

For *C. capitata*, a total of 345 samples was used. After outlier removal, 272 samples were used for model development, which exhibited overall classification accuracies exceeding 95% across both classes and for both tested KNN distance values. Despite this, the prediction capability of the models resulted in very low predictive performance (Table 5).

### 3.3. Oranges

**Figure 7:**
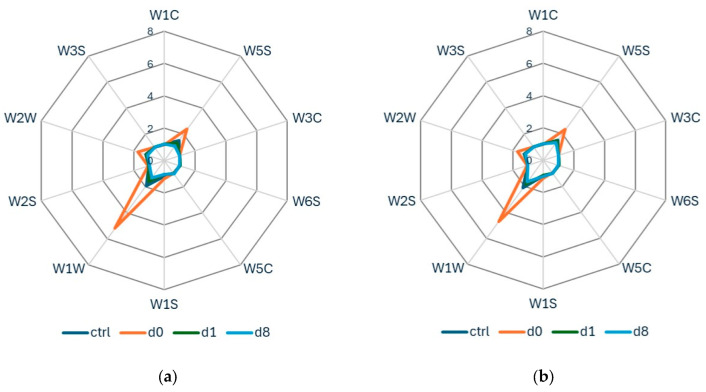
Radar plots of e-nose sensor response values for the Valencia orange variety (**a**) after infestation by *C. capitata* and (**b**) *B. zonata*.

For oranges, the radar plots indicated that sensor W1W had the highest average response, followed by W5S, across all combinations of variety and FF. Differences were recorded at different time points of infestation. Immediately after oviposition, the W1W sensor exhibited the highest response. Two characteristic radar plots are shown in Figure 7.

**Figure 8:**
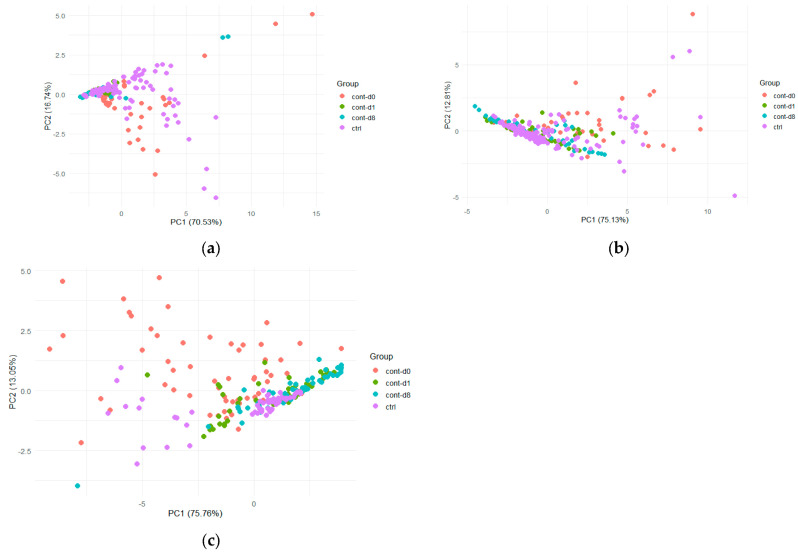
PCA score plots using the first two PCs (principal components) for all samples of Valencia oranges, analyzed and divided into six classes for E-nose data acquired after infestation by (**a**) *B. dorsalis* and (**b**) *B. zonata* and into four classes for E-nose data acquired after infestation by *C. capitata* (**c**) stored at 25 °C.

In Figure 8 the PCA score plots for Valencia oranges infested by the two *Bactrocera* species and *C. capitata* are shown.

Washington Navel

*B. dorsalis*

The initial dataset consisted of 287 samples (Table 1), which decreased to 194 for further manipulation and model construction. Models using KNN with k = 1 and k = 3 were developed by classifying samples into either four or six categories for the Washington navel variety (Table 6). For the six-class model, the accuracy rate was 68%. The same tendency was observed with the four-class model, with the overall predictive capability of the model at 82% for KNN = 3 and 81% for KNN = 1, but the accuracy within the class categories was considerably lower (Supplementary Materials).

*B. zonata*

A total of 296 samples were in the initial dataset for *B. zonata*, which reached 183 samples after outlier removal. The overall classification rates in the prediction set for the six-class models were slightly above 60%. The four-class models had improved predictive performance; however, the overall classification accuracy did not exceed 75% for either KNN = 1 or KNN = 3.

*C. capitata*

Similarly, for *C. capitata*, the initial set consisted of 280 samples, of which 115 samples were used for model construction. The overall predictive capability of the model was 56% for both KNN values used (KNN 1 and 3). The four-class strategy slightly improved the model’s performance at K = 3 and K = 1 compared to the six-class models. The overall predictive capability of the model was 69% for KNN = 3 and 75% for KNN = 1.

Valencia

For Valencia oranges, models based on six and four classes were explored with KNN 1 and 3 (Table 7), except for *C. capitata*, for which only four-class models were constructed. The KNN models were evaluated for all three fruit fly species at 25 °C. The overall rates for calibration and cross-validation ranged from 67 to 88%, while the overall prediction accuracy ranged from 56 to 82%. However, the accuracy within the class categories was considerably lower (Supplementary Materials).

*B. dorsalis*

For *B. dorsalis*, a total of 417 samples were acquired, of which 204 were further used for model construction. Applying the six-class approach, model performance was poor, with a prediction accuracy of 65% for both KNN 1 and 3. The use of the four-class approach improved overall prediction accuracy to 80%.

*B. zonata*

For *B. zonata*, the initial dataset consisted of 391 samples. A first round of outlier removal was performed, and the dataset was reduced to 289 samples. No acceptable results were obtained. A second round of outlier removal was performed, considerably decreasing the total number (n = 205). The six-class models achieved only 49% predictive accuracy for both KNN = 1 and KNN = 3. The four-class models showed slight improvement, reaching 58% accuracy for KNN = 3 and 68% for KNN = 1.

*C. capitata*

In the case of *C. capitata*, a total of 421 samples were acquired. After the outlier removal process (mainly from control samples), the number of samples decreased to 223. The six-class model construction was not feasible due to an insufficient number of control samples. A model with four classes achieved 74% prediction accuracy for both KNN = 1 and KNN = 3.

### 3.4. Mandarins

**Figure 9:**
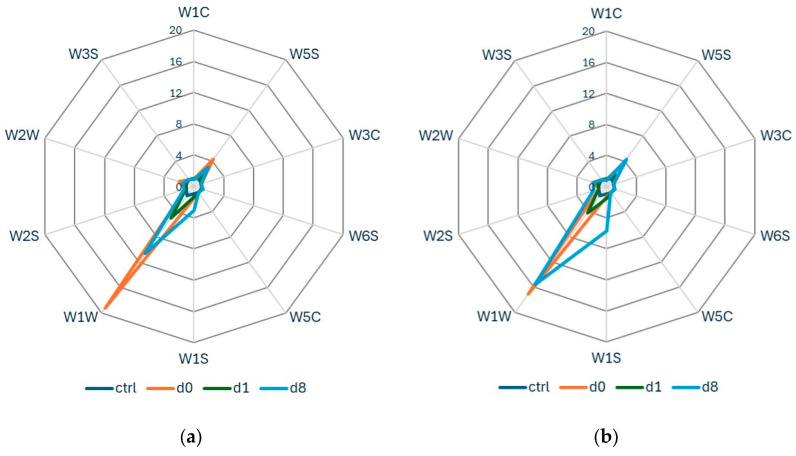
Radar plots of e-nose sensor response values for mandarins (**a**) after infestation by *C. capitata* and (**b**) *B. zonata*.

During autumn and winter 2022, data were collected from mandarin fruits, variety clementine. Fruits were infested with *B. zonata* and *C. capitata* and stored at 25 °C. The radar plots showed that sensor W1W exhibited the highest average response, followed by W5S, for both fruit fly species (Figure 9). W1W also had the highest response in the orange datasets, implying the sensitivity of this sensor to terpenes [25], which are the dominant volatile compounds in citrus [17].

**Figure 10:**
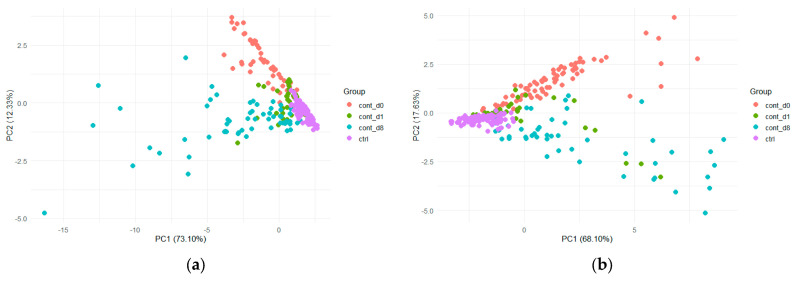
PCA score plots performed on the first two PCs (principal components) for mandarin samples analyzed and divided into four classes for E-nose data acquired after infestation by (**a**) *B. zonata* and (**b**) *C. capitata* and stored at 25 °C.

In Figure 10, the PCA score plots for mandarins infested by *B. zonata* and *C. capitata* are shown. Samples infested by both FF species can be distinguished according to infestation status and time along PC2. For both species, the loading plots revealed that sensors W1W, W2W, W3S and W5S characterize the signal of samples collected at the beginning of contamination, whereas samples 8 days after infestation are characterized by the signal of W1S, W2S and W6S (Supplementary Materials).

*B. zonata*

For *B. zonata*, a total of 343 samples were acquired and used for model construction. Only the four-class model using KNN = 3 could be constructed. A new independent set of 142 samples was used for prediction, and a predictive accuracy of 78% was achieved, indicating moderate performance (Table 8).

*C. capitata*

The dataset for calibration and cross-validation consisted of 414 samples. Models using KNN with k = 1 and k = 3 were developed by classifying samples into four classes (Table 8). An independent dataset (n = 175) was acquired for prediction, and the predictive accuracy for the model with KNN = 3 was 72%, but it was tremendously decreased (32%) when a k distance of 1 was used.

## 4. Discussion

Plants, including fruits, following oviposition and larval development, exhibit variation in VOC emitted as a response to both oviposition and herbivory [42,43]. Typical compounds reported include terpenes, esters, alcohols, aldehydes, and ketones, which are involved in fruit aroma and plant defense signaling. Peach aroma consists of esters, lactones, and terpenes [44], while esters and *(E,E)-α*-farnesene are the main compounds in apple aroma [15]. We recently reported that the main compounds detected in the headspace of orange fruits are mainly terpenes and esters [17]. Following infestation by *Tephritidae*, the volatile profile of fruits is altered through either quantitative changes in emitted VOCs, shifts in the relative proportions of existing compounds, and/or qualitative changes involving the induction of new volatile compounds [7,14,15,16,17,18,19,20]. For instance, increases in and simultaneous presence of *E-β*-ocimene, DMNT, hexyl butanoate, butyl hexanoate, and hexyl hexanoate are correlated with the early stage of infestation in *C. capitata* oranges [17]. Wen et al. [33] claimed that hexanol could be an indicator for citrus fruit infested by *B. dorsalis.* Apples infested by *Bactrocera tryoni* emitted lower total amounts of VOCs than non-infested apples, while certain ethyl esters have been associated with early larval development [15].

The present study is the first effort to explore the potential of a commercially available e-nose for the discrimination of fruit fly infestation. Our aim was to exploit the response of fruits to herbivory to develop a rapid and reliable detection method for fruit fly infestation during routine inspections. We chose to explore the capabilities of the commercially available PEN3 E-nose, which is fabricated for general purposes, such as environmental samples and the food industry [21,22]. We managed to construct models based on each fruit fly and fruit combination with two KNN options (either one or three). Our models showed varying predictive capabilities across fruit species combinations. The apple–*B. zonata* and apple–*C. capitata* combinations yielded the highest discrimination performance during training, suggesting that VOC changes in these systems are more distinct. However, external validation trials, using new, independent samples, showed reduced accuracy, likely due to year-to-year variability in VOC emission profiles.

Variability in VOC profiles is well documented and can arise from differences in cultivars, infestation levels, storage conditions, and abiotic conditions during the growing season, like temperature, humidity, etc. [17,18,20,22,40,44]. For instance, differences in emitted VOCs among peach varieties have been documented in the literature [44], and variations in VOCs for the same variety have also been reported among different studies, indicating a possible effect due to growth conditions, which could be attributed to the actual growth conditions or to seasonal environmental differences among locations [17,20]. It is challenging to incorporate all these variations into the developed model of an e-nose. Although we used a large dataset for each treatment in an effort to capture VOC variability, it seems that training the e-nose might require a much larger number of replicates. We included variation in our treatments whenever possible, such as storage time, temperature and cultivar. Indeed, there were differences in odor profiles among the cultivars and temperatures used for storage conditions, which affected the model’s discriminative capability. It is likely, though, that seasonal variation is quite large, and although certain models incorporated data from more than one season, the overall variability was not integrated in the constructed model. That would require extensive sampling across seasons to increase sample size and eventually include the maximum variability in the model.

Another possible explanation is the use of the entire VOC profile for training the e-nose and ultimately for model construction. However, the differences among the treatments used for infested and non-infested fruits, as well as among fruit fly species and fruit combination, were most likely due to quantitative or qualitative differences of specific chemical compounds [45]. The reduced predictive performance during external validation probably reflects this biological and environmental diversity, highlighting the importance of using multi-season datasets and developing adaptive calibration models. Several cases have focused on the use of a specific e-nose for the detection of fungi on postharvest products [30] or plant diseases [31]. There are fewer cases where the PEN3 e-nose has been used for insect detection [46]. In almost all cases used so far, the studies were conducted in controlled laboratory settings with a limited amount of data, which is more likely to result in precise calibration and repeatability. Furthermore, validation of the models constructed based on an external dataset is usually lacking [47]. Moreover, higher model accuracy is often achieved with custom-built sensor arrays, whereas e-noses incorporating multiple MOSs produce large multivariate response datasets, which may result in poor model performance and classification accuracy [47]. In our study, across all fruit datasets, the W1W sensor showed the highest average response, followed by W2W, W5S, and W1S. These sensors have also been linked to e-nose responses to the floral scent of cut lilies, where the dominant compounds were terpenes, sesquiterpenes, benzenoids/phenylpropanoids, and fatty acid derivatives [48]. While e-nose sensors are capable of detecting a wide range of VOCs, they often have difficulty in distinguishing between compounds with similar chemical structures, and interference from other gases can introduce noise, reduce their selectivity and sometimes cause false positives or false negatives [22]. Not all sensor responses are equally informative; only a subset may be truly important for accurate classification. Including redundant sensor responses can even lead to overfitting, affecting a model’s predictive capability [22,47]. Moreover, the sensitivity and specificity of an odor-based sensor can depend on the product being tested (for example, the cultivar), its condition (age and storage time), and the type of herbivore, along with other factors. Previous studies have shown that the same sensor array, the commercial Cyranose 320 consisting of 32 sensors, can successfully detect rice weevil and red flour beetle infestation in rice, but it fails to identify others, such as the lesser grain borer [37]. Custom-built sensor arrays used to detect varying densities of *Ephestia kuehniella* on white flour demonstrated greater discrimination accuracy for certain larval instars compared with others [38].

In our study, the actual capacity of the models is proved through a thorough validation process in which new, external and unclassified samples are used, a methodological advancement over earlier reports. We validated the most promising models constructed with new datasets to verify their discriminative potential. Although our validation models did not achieve high accuracy, this study highlights key challenges and provides a valuable starting point for developing more robust, transferable models. Previous studies often refrained from validating e-nose performance with independent datasets, which we largely employed in our study. Thus, we showed that this is a necessary step in the process for obtaining a clear understanding of the limitations and the actual potential of the developed tools.

Nevertheless, the importance of plant-emitted VOCs in modern agriculture is continuously acknowledged. Not only for improving efficiency in border inspections to avoid the introduction of invasive species with detrimental effects on agricultural systems, but also for sustainable management in the field. There are several ongoing efforts to utilize available technology to the maximum extent possible to improve plant disease and pest detection in the field for resilient and sustainable agriculture [49,50]. The global trend, as well as our future aim, is to focus on specific VOC biomarkers identified using GC that, through chemometric analysis, are shown to be responsible for the differences among infested and non-infested commodities. This approach is of primary importance for fast, affordable and reliable detection of infestation at an early stage before visible signs appear. Optimizing sensor features and combining e-nose data with machine learning methods may further enhance predictive performance. Overall, integrating e-nose sensing with VOC profiling shows strong potential for developing rapid, affordable, and field-ready tools for early detection of fruit fly infestations.

## Figures and Tables

**Table 1:** The classes and corresponding treatments applied for the model development for each fruit and for different temperatures, as well as the number of replicates for each treatment, per fruit, per year (Y1 = 1st year, Y2 = 2nd year), per variety (W = Washington navel, V = Valencia).

Fruit	Temperature	Classes	Treatments	*B. dorsalis*	*B. zonata*	*C. capitata*
Peaches	20 °C	4	1. Control (control d0 and control d1)	90		119
			2. mature control (control d7)	32		55
			3. d0 and d1	93		121
			4. d7	42		55
		6	1. Control d0	45		61
			2. Control d1	45		58
			3. Control d7	32		55
			4. d0	48		61
			5. d1	45		60
			6. d7	42		55
	25 °C	4	1. Control (control d0 and control d1)	113		64
			2. mature control (control d4)	62		46
			3. d0 and d1	103		113
			4. d4–d5	57		58
		6	1. Control d0	54		48
			2. Control d1	59		46
			3. Control d4	62		46
			4. d0	50		59
			5. d1	53		54
			6. d4	57		58
Apples	6 °C	3		Y1–Y2	Y1	Y1–Y2
			1. Control (all control samples)	126–42	126	126–42
			2. d0	68–39	37	65–33
			3. d15	60–39	38	46–33
		4		Y1–Y2	Y1	Y1–Y2
			1. Control d0	70–21	70	70–21
			2. Control d15	56–21	56	56–21
			3. d0	68–39	37	65–33
			4. d15	60–39	38	46–33
Oranges	25 °C	4		W–V	W–V	W–V
			1. Control (all samples)	162–231	176–231	162–231
			2. d0	46–64	46–54	40–63
			3. d1	41–64	40–54	40–62
			4. d8	39–58	35–52	38–66
		6		W–V	W–V	W–V
			1. Control d0	57–75	59–75	54–75
			2. Control d1	55–82	65–82	50–82
			3. Control d8	50–74	52–74	58–74
			4. d0	46–64	46–54	40–63
			5. d1	41–64	40–54	40–62
			6. d8	39–58	35–52	38–66
Mandarins	25 °C	4	1. Control (all samples)	230		230
			2. d0	65		94
			3. d1	70		88
			4. d8	60		94

**Table 2:** The overall classification rates (% percentage) for the calibration (cal), cross-validation (CV), and prediction sets for all peach models constructed for *Bactrocera zonata* and *Ceratitis capitata* at 20 °C and 25 °C.

Fruit Fly	Storage Temperature	Classes	KNN	Cal (%)	CV (%)	Prediction (%)
*B. zonata*	20	6	3	50	58	67
		6	1	50	58	67
		4	3	64	63	74
		4	1	80	81	85
	25	6	3	71	73	57
		6	1	71	73	57
		4	3	76	79	59
		4	1	84	84	65
*C. capitata*	20	6	3	64	68	71
		6	1	64	69	71
		4	3	72	76	80
		4	1	83	85	85
	25	6	3	61	69	61
		6	1	82	82	71
		4	3	63	69	69
		4	1	83	85	75

**Table 3:** The overall classification rates (% percentage) for the calibration (cal), cross-validation (CV), and prediction sets for all apple models constructed for *B. dorsalis*, *B. zonata*, and *C. capitata* at 6 °C in 2022.

Fruit Fly Species	Classes	KNN	Cal (%)	CV (%)	Prediction (%)
*B. dorsalis*	4	3	80	80	80
	4	1	83	85	88
	3	3	82	82	83
	3	1	85	85	91
*B. zonata*	4	3	67	69	69
	4	1	67	69	69
	3	3	72	73	71
	3	1	72	76	84
*C. capitata*	4	3	73	74	79
	4	1	78	80	85
	3	3	74	76	80
	3	1	80	82	86

**Table 4:** The overall classification rates (% percentage) for the calibration (cal), cross-validation (CV), and prediction sets for all apple models constructed for *B. dorsalis* and *C. capitata* at 6 °C, with data for the year 2023.

Fruit Fly Species	Classes	KNN	Cal (%)	CV (%)	Prediction (%)
*B. dorsalis*	4	3	69	71	81
	4	1	67	75	85
	3	3	92	94	92
	3	1	92	96	92
*C. capitata*	4	3	91	89	79
	4	1	89	86	79
	3	3	95	93	97
	3	1	91	91	97

**Table 5:** The overall classification rates (% percentage) for the calibration (cal), cross-validation (CV), and prediction sets for all apple models constructed for *B. dorsalis*, and *C. capitata* at 6 °C, with data for the years 2022 and 2023.

Fruit Fly Species	Classes	KNN	Cal (%)	CV (%)	Prediction (%)
*B. dorsalis*	4	3	91	91	21
	4	1	92	92	21
	3	3	94	93	47
	3	1	95	95	47
*C. capitata*	4	3	95	95	20
	4	1	95	99	18
	3	3	98	97	42
	3	1	99	99	38

**Table 6:** The overall classification rates (% percentage) for the calibration (cal), cross-validation (CV), and prediction sets for all Washington avel orange models constructed for *Bactrocera dorsalis*, *Bactrocera zonata*, and *Ceratitis capitata* at 25 °C.

Fruit Fly Species	Classes	KNN	Cal (%)	CV (%)	Prediction (%)
*B. dorsalis*	6	3	67	75	68
	6	1	79	79	74
	4	3	73	78	82
	4	1	83	83	81
*B. zonata*	6	3	66	67	61
	6	1	78	72	66
	4	3	76	75	75
	4	1	80	81	75
*C. capitata*	6	3	70	70	56
	6	1	70	70	56
	4	3	79	77	69
	4	1	88	87	75

**Table 7:** The overall classification rates (% percentage) for the calibration (cal), cross-validation (CV), and prediction sets for all Valencia orange models constructed for *Bactrocera dorsalis*, *Bactrocera zonata*, and *Ceratitis capitata* at 25 °C.

Fruit Fly Species	Classes	KNN	Cal (%)	CV (%)	Prediction (%)
*B. dorsalis*	6	3	73	77	65
	6	1	73	77	65
	4	3	84	84	80
	4	1	84	84	80
*B. zonata*	6	3	56	64	49
	6	1	56	64	49
	4	3	65	71	58
	4	1	75	77	68
*C. capitata*	6	3	-	-	-
	6	1	-	-	-
	4	3	63	67	74
	4	1	63	67	74

**Table 8:** The overall classification rates (% percentage) for the calibration (cal), cross-validation (CV), and prediction sets for all mandarin fruit models, variety clementine, constructed for *Bactrocera zonata* and *Ceratitis capitata* at 25 °C.

Fruit Fly Species	Classes	KNN	Cal (%)	CV (%)	Prediction (%)
*B. zonata*	4	3	88	88	78
	4	1	-	-	-
*C. capitata*	4	3	87	88	77
	4	1	90	95	32

## Data Availability

The original contributions presented in this study are included in the article/Supplementary Materials. Further inquiries can be directed to the corresponding author.

## Supplementary Materials

The following supporting information can be downloaded at https://www.mdpi.com/article/10.3390/insects17040429/s1: Table S1: MOS Sensor names and characteristics; Table S2: Figure captions for Figures S1–S9; Figures S1–S9: Loading plots for the corresponding PCA plots in Figure 3, Figure 6, Figure 8 and Figure 10; Excel File S1: Confusion matrices for the peach fruit model; Excel File S2: Confusion matrices for the apple fruit model; Excel File S3: Confusion matrices for the orange fruit model; Excel File S4: Confusion matrices for the mandarin fruit model.
