# Supplementary material for: Application of an Electronic Nose for Early Detection of Tephritidae Infestation in Fruits
Source: Insects. 2026 Apr 16;17(4):429. doi: 10.3390/insects17040429 (PMC13116407; doi:10.3390/insects17040429)
Supplement: Supplementary file 1 [file insects-17-00429-s001.zip › Table S1.pdf]

Table S1. MOS Sensor's names and characteristics

No of sensors	Sensor name	Sensor description and sensitivities
1	**W1C**	Aromatic organic compounds
2	**W5S**	Very sensitive, broad range sensitivity, reacts to nitrogen oxides, very sensitive with negative signal
3	**W3C**	Ammonia, also used as sensor for aromatic compounds
4	**W6S**	Mainly hydrogen gas
5	**W5C**	Alkanes, aromatic compounds, and non-polar organic compounds
6	**W1S**	Sensitive to methane. Broad range of organic compounds detected
7	**W1W**	Detects inorganic sulfur compounds, e.g. H2S. Also sensitive to many terpenes and sulfur containing organic compounds
8	**W2S**	Detects alcohol, partially sensitive to aromatic compounds, broad range
9	**W2W**	Aromatic compounds, inorganic sulfur and organic compounds
10	**W3S**	Reacts to high concentrations of methane and aliphatic organic compounds
