# Supplementary material for: Application of an Electronic Nose for Early Detection of Tephritidae Infestation in Fruits
Source: Insects. 2026 Apr 16;17(4):429. doi: 10.3390/insects17040429 (PMC13116407; doi:10.3390/insects17040429)
Supplement: Supplementary file 1 [file insects-17-00429-s001.zip › Table S2.pdf]

Table S2. Figure caption for figures S1-S9

S1	Loadings_Fig. 3A
S2	Loadings_Fig. 3B
S3	Loadings_Fig. 6A
S4	Loadings_Fig. 6B
S5	Loadings_Fig. 8A
S6	Loadings_Fig. 8B
S7	Loadings_Fig. 8C
S8	Loadings_Fig. 10A
S9	Loadings_Fig. 10B
