# Supplementary figures and images for: Application of an Electronic Nose for Early Detection of Tephritidae Infestation in Fruits
Source: Insects. 2026 Apr 16;17(4):429. doi: 10.3390/insects17040429 (PMC13116407; doi:10.3390/insects17040429)

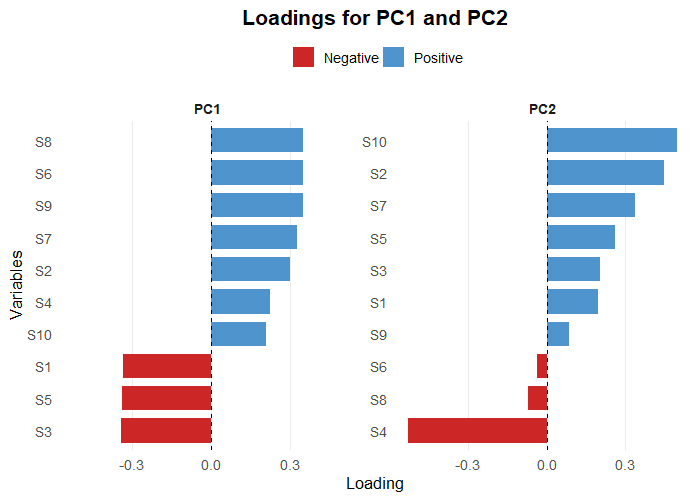

Supplement: Supplementary file 1 [file insects-17-00429-s001.zip › S1. Loadings_Fig. 3A.png]

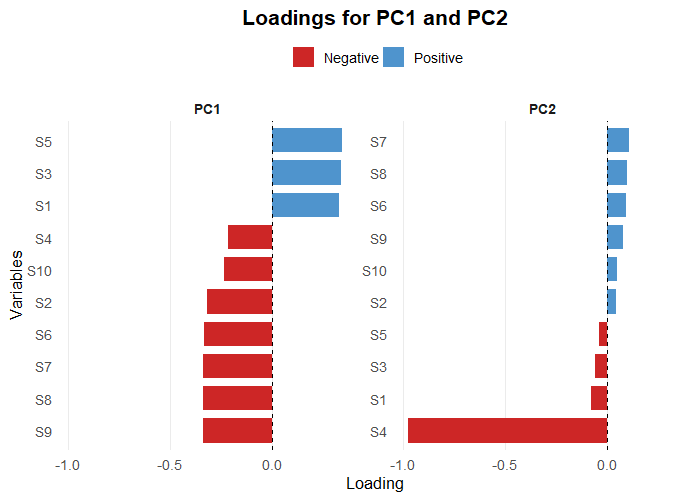

Supplement: Supplementary file 1 [file insects-17-00429-s001.zip › S2. Loadings_Fig. 3B.png]

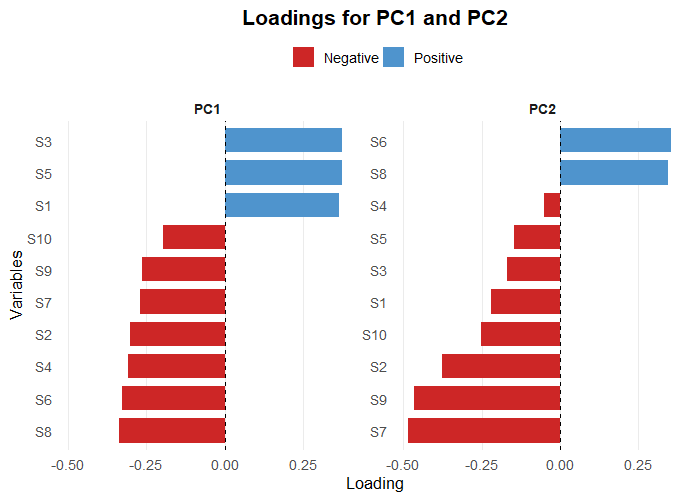

Supplement: Supplementary file 1 [file insects-17-00429-s001.zip › S3. Loadings_Fig. 6A.png]

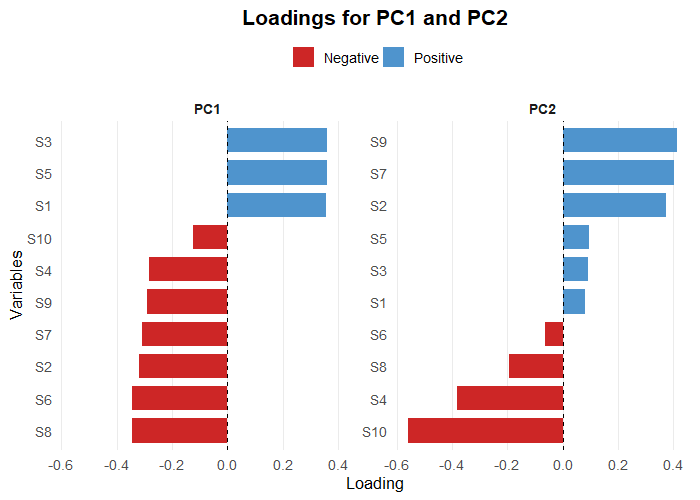

Supplement: Supplementary file 1 [file insects-17-00429-s001.zip › S4. Loadings_Fig. 6B.png]

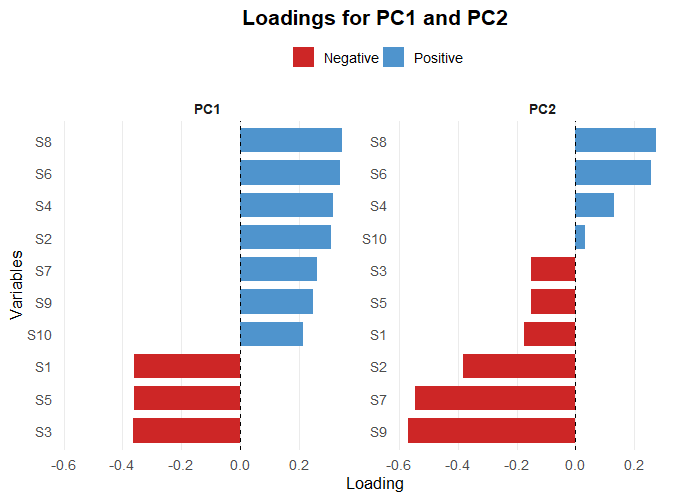

Supplement: Supplementary file 1 [file insects-17-00429-s001.zip › S5. Loadings_Fig. 8A.png]

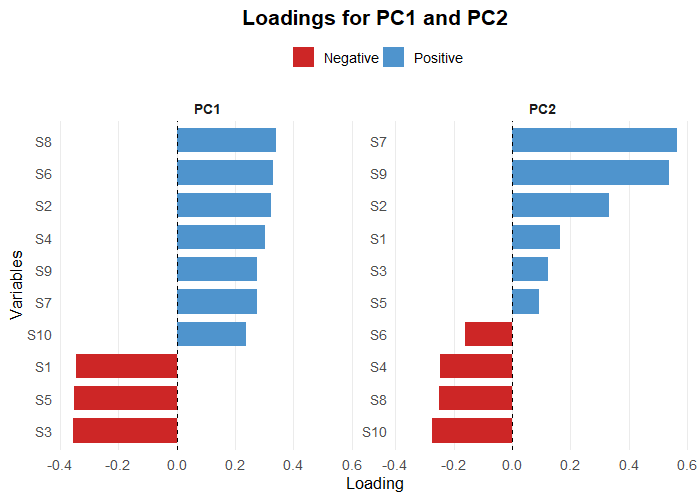

Supplement: Supplementary file 1 [file insects-17-00429-s001.zip › S6. Loadings_Fig. 8B.png]

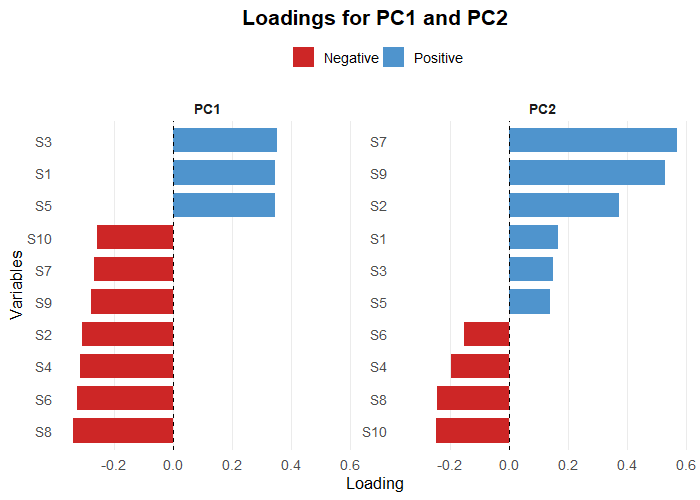

Supplement: Supplementary file 1 [file insects-17-00429-s001.zip › S7. Loadings_Fig. 8C.png]

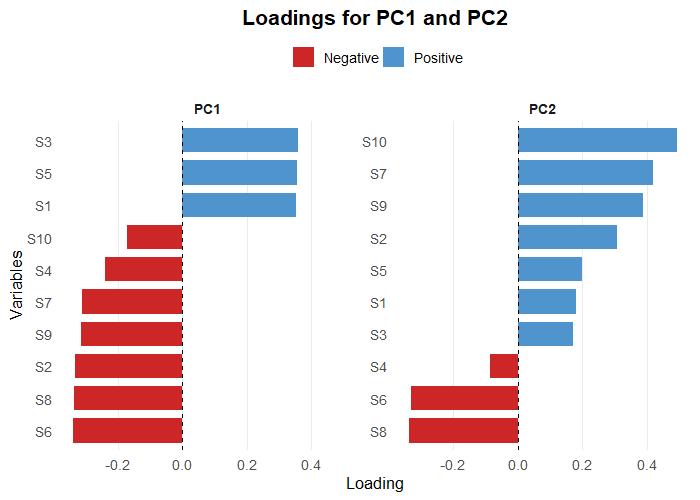

Supplement: Supplementary file 1 [file insects-17-00429-s001.zip › S8. Loadinds_Fig. 10A.png]

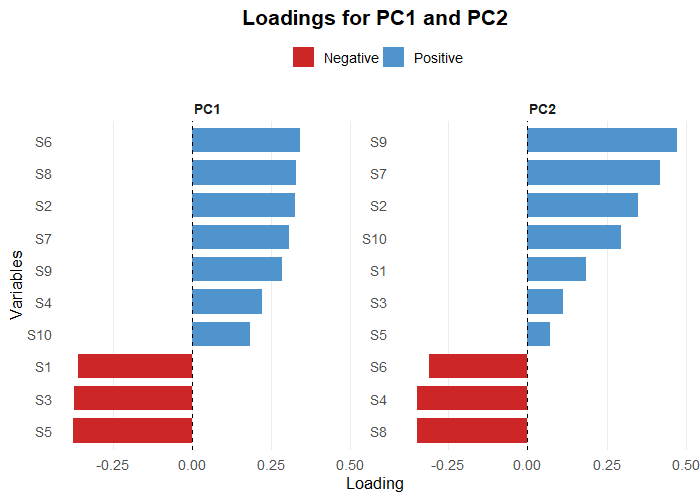

Supplement: Supplementary file 1 [file insects-17-00429-s001.zip › S9. Loadings_Fig. 10B.png]
